# Metasurfaces for next-generation wireless communication systems

**DOI:** 10.1093/nsr/nwad140

**Published:** 2023-05-15

**Authors:** Younes Ra’di, Nikita Nefedkin, Petar Popovski, Andrea Alù

**Affiliations:** Department of Electrical Engineering and Computer Science, Syracuse University, USA; Photonics Initiative, Advanced Science Research Center, City University of New York, USA; Department of Electronic Systems, Aalborg University, Denmark; Photonics Initiative, Advanced Science Research Center, City University of New York, USA; Physics Program, Graduate Center, City University of New York, USA

## Abstract

Tailored time variations, nonlinearities and active elements can endow metasurfaces with unique opportunities for next-generation wireless communication systems, enriching the growing platform of reconfigurable intelligent surfaces.

The pace of development in the world of 5G communication systems has proven to be much more demanding than previous generations, with 5G-Advanced seemingly around the corner [1]. Extensive research is already underway to structure the next generation of wireless systems (i.e. 6G), which may potentially enable an unprecedented level of human–machine interaction [2]. Naturally, such a fast-paced development requires exponential growth in system requirements, both in terms of exploration of new paradigms for hardware and for the underlying communication protocols. The idea is to move towards robust and highly reconfigurable wireless systems going well beyond the limits of current technology, putting yet harsher constraints on different aspects of these systems including data rates, reliability, latency and massiveness.

Despite these ever-growing developments and demands for wireless communications, the wireless propagation environment has so far been largely determined by nature, making it the most challenging portion of the communication system to control, and thereby requiring the end devices to adapt their communication strategies to uncontrollable and fast-changing environments. Reconfigurable intelligent surfaces (RISs) have recently undertaken a key role in tackling these challenges by providing a reconfigurable hardware platform enabling dynamic control over their response to incoming waves [3,4]. Despite extensive research progress and several implementations of RISs in communication systems, we believe that their potential and impact can be significantly augmented by catering new and unconventional electromagnetic responses unveiled by recent developments in the physics/electromagnetics communities in the context of metasurfaces.

So far, linearity, passivity and reciprocity have been the basic premises in the design of modern communications systems. The RIS concept and its generalizations have the potential to alter these assumptions, augment the set of functionalities that these surfaces can provide and lead to new communication methods and protocols. Getting better control over the changing environment requires many more degrees of freedom in their design. Metasurfaces consisting of active, non-liner and time-varying elements [5–8] offer a plethora of new opportunities for wireless communication systems, inspiring novel communication models and wireless architectures (Fig. 1). In this context, mmWave/THz metasurfaces with highly tunable elements can be reconfigured in real time pixel by pixel to respond to changes in the background and in user locations. In addition, the inclusion of gain and active elements may compensate for propagation loss, important as the carrier frequencies grow to accommodate broader bandwidths and larger data rates. Time variations across the metasurface aperture may also realize non-reciprocal responses, and tailored non-linearities may enable frequency mixing and tailored signal processing and manipulation. Overall, these elements can extensively enrich what is possible with current RIS implementations. Such intelligent metasurfaces will enable unconventional wave–RIS interactions, playing key roles in the structure of future communication architectures (Fig. 1).

**Figure 1.**
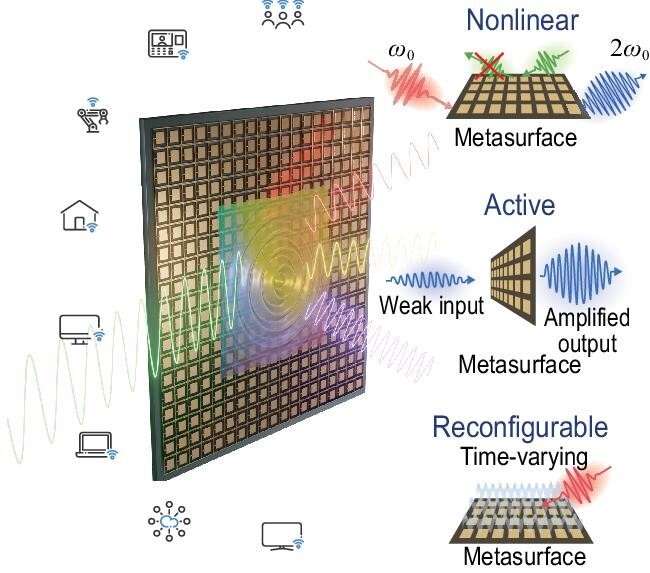
Envisioned metasurface platform to enable non-linear, active and time-varying operations for next-generation wireless communication systems.

In this context, recent developments in the field of metasurfaces have started to showcase the rich potential of implementing complex transformations of the incoming signals, both in terms of the spatial content, the frequency content and the angular spectrum. These features largely expand what is possible in state-of-the-art RISs used in current communication systems. Advances in non-linear, active, reconfigurable and time-varying metasurfaces indeed promise a large leap forward in wave manipulation over the next generation of communication systems. Some of this progress has started to trickle down from the field of applied electromagnetics and physics to the field of wireless communications and electronic engineering, with the goal of impacting real-life systems. A few proof-of-concept realizations of these forms of intelligent metasurfaces have recently been reported, however with limited considerations of the relevant challenges in real-life communication systems and relevant metrics of performance required to impact these systems (e.g. [9,10]). A stronger synergy and a more in-depth mutual understanding between the metasurface and wireless communication communities may be able to leverage the full potential of recent progress in the field of metasurfaces for next-generation communication systems.

For instance, non-reciprocal responses will bring new opportunities for totally asymmetric responses, enabling full-duplex operations and compensation of Doppler shifts in the case of moving users. Highly programmable metasurfaces may optimally reconfigure their operation in response to changes in the environment and user positions, exploiting tailored pilot signals probing the environment. Furthermore, space-time-modulated metasurfaces can bring a whole new set of functionalities to conventional RISs, including parametric phenomena and wave mixing. For instance, efficient broadband phase conjugation based on parametric mixing may be used to realize efficient channel estimation and analog signal processing with low latency and reduced energy requirements. Time-modulated and non-linear metasurfaces will enable transformation of the frequency spectrum of the outgoing signals with respect to the input signals, which may be exploited to optimally use the limited bandwidth in the frequency spectrum (see insets in Fig. 1). As an additional opportunity, metasurfaces offer an agile platform for analog signal processing, leveraging advanced dispersion engineering through their non-local response to impart on the impinging signals mathematical operations of choice. These advanced metasurfaces will be extremely beneficial for wireless communication based on ambient backscatter. Furthermore, these surfaces can facilitate the integration of communication, sensing and computing systems, which is an emerging platform in new generations of communication systems. The emergence of advanced communication systems requires denser nodes, which as a result calls for careful considerations of electromagnetic compatibility. These metasurfaces can enable highly efficient spectral/spatial shielding.

Such a platform appears ideal to bring together wave physicists and electrical and communication engineers, offering a considerable opportunity for mutual awareness and collaborative efforts between these two communities. The metasurface community needs to be more frequently exposed to opportunities in the context of future wireless communication and state-of-the-art challenges that they can address. On the other hand, the wireless communication community should be made aware of the extent of the functionalities that advanced metasurfaces have been recently enabling, especially when considering the direct integration of active, non-linear and time-modulated elements.

New wireless architectures and new deployment schemes will be needed to exploit the described opportunities offered by development in the area of metasurfaces. New frequency regulations may need to be designed to facilitate employing the unique flexibility of advanced metasurfaces, which enable the modification, at will, of the frequency spectrum and the bandwidth of wireless signals. Employing time-modulated metasurfaces will also require progress in the power handling of elements that can modulate the input signals at GHz frequencies. The use of complex control networks with engineered feedback will largely enhance the number of degrees of freedom, and moving beyond complementary metal-oxide semiconductor (CMOS)-based circuits, for instance using gallium nitride elements, may enable stronger tailored non-linearities and better power handling. At higher frequencies (e.g. mm Waves and waves at terahertz frequencies), the required tunability can be achieved using liquid crystals, phase-change materials and micro-electro-mechanical resonators. However, new approaches need to be explored to enable faster mechanisms of tunability in these structures.

Initiating dialogue and research on the applicability of these metasurfaces in real-life communication systems is the main motivation for this Perspective. In general, a close synergy between wireless communication engineers and electromagnetic engineers over time may be able to take full advantage of the metasurface platform for specific frequencies and target operations.
